# Aberrant alternative splicing of purinergic receptor P2RX4 prevents sensitivity towards combinatorial treatment in colorectal and pancreatic cancer

**DOI:** 10.1002/path.70082

**Published:** 2026-06-03

**Authors:** Christoph Steup, Julian Dosch, Christopher Dietz‐Fricke, Sara Khosraviseftejani, Esther Engel, Adalbert F de Valk, Dominic Menger, Patrick Welker, Peter J Wild, Kilian B Kennel, Maja Kantlehner, Paul K Ziegler, Florian R Greten

**Affiliations:** ^1^ Institute for Tumor Biology and Experimental Therapy Georg‐Speyer‐Haus Frankfurt am Main Germany; ^2^ Goethe University Frankfurt, Department of Medicine I University Hospital Frankfurt Frankfurt am Main Germany; ^3^ Frankfurt Cancer Institute, Goethe University Frankfurt Frankfurt am Main Germany; ^4^ Department of Gastroenterology, Hepatology, Infectious diseases and Endocrinology Hannover Medical School Hannover Germany; ^5^ Goethe University Frankfurt, Dr. Senckenberg Institutes of Pathology and Human Genetics (SPH), University Frankfurt, University Hospital Frankfurt Frankfurt am Main Germany; ^6^ German Cancer Consortium (DKTK) and German Cancer Research Center (DKFZ) Heidelberg Germany

**Keywords:** therapy resistance, P2RX4, colorectal cancer, pancreatic cancer, alternative splicing

## Abstract

Recently, we suggested the combination of chemotherapy and P2RX4 inhibition as a promising novel therapeutic approach for P2RX4‐expressing epithelial tumors to prevent paracrine resistance. Here, we aimed to assess whether determining *P2RX4* expression status in colorectal and pancreatic cancer patients would allow stratification of potentially responsive patients. Therefore, *P2RX4* expression levels were determined by RNA sequencing and immunohistochemistry. Subcellular localization of P2RX4 isoforms was analyzed in HeLa cells and patient‐derived tumor organoids. In contrast to its RNA expression profile, P2RX4 protein levels exhibited differential regulation in human colorectal and pancreatic cancer epithelia due to alternative splicing. Interpatient heterogeneity was greater in colorectal cancer than in pancreatic cancer. Notably, these variations in expression did not correlate with overall patient survival. Alternative *P2RX4* transcripts gave rise to functionally distinct protein isoforms that differed in subcellular localization and total protein abundance. Only the correctly spliced, canonical P2RX4 isoform was localized to the plasma membrane and was capable of mediating downstream signaling. Accordingly, P2RX4 inhibition in combination with chemotherapy was effective exclusively in patient‐derived tumor organoids expressing the canonical *P2RX4* transcript. In summary, immunohistochemical, but not transcriptomic, assessment of P2RX4 expression enabled the prediction of sensitivity to combinatorial treatment and facilitated the identification of patients who may benefit from P2RX4 inhibition during chemotherapy. Given the lower degree of heterogeneity observed in pancreatic cancer, this tumor entity may represent a promising candidate for early‐phase clinical evaluation of chemotherapy combined with P2RX4 inhibition. © 2026 The Author(s). *The Journal of Pathology* published by John Wiley & Sons Ltd on behalf of The Pathological Society of Great Britain and Ireland.

## Introduction

Primary or secondary therapy resistance is a major challenge in the treatment of advanced cancers [1] and accounts for up to 90% of cancer‐related deaths [2, 3]. Beyond resistance driven by the acquisition of genetic mutations and clonal evolution, transient, non‐genetic transcriptional adaptation has been increasingly recognized as a key contributor to therapy resistance [4, 5, 6, 7, 8, 9, 10]. Recently, we identified a paracrine mechanism of therapy resistance in colorectal cancer (CRC) and pancreatic ductal adenocarcinoma (PDAC) cells which rendered surviving cells resistant to the cytotoxic treatment. Chemotherapy‐induced tumor cell death led to the release of reactive oxygen species (ROS) and extracellular adenosine triphosphate (ATP). While ROS triggered a pro‐apoptotic program in neighboring tumor epithelial cells, ATP counteracted this effect by engaging a P2RX4 receptor (P2RX4) and mTOR‐mediated pro‐survival program. Consequently, concomitant P2RX4 or mTOR inhibition during chemotherapy led to rapid tumor regression that was not observed when the respective inhibitors were applied individually. Conversely, ROS scavenging prevented cancer cells from becoming dependent on mTOR activation [11]. Therefore, dying cancer cells profoundly ‘rewire’ signaling pathways in their surviving neighbors, establishing a new dependency on anti‐apoptotic programs, thereby creating a novel opportunity for combining standard therapy with P2RX4 inhibition in P2RX4‐expressing epithelial tumors, such as CRC and PDAC.

P2RX4 belongs to the family of ATP‐gated cation channels and is assembled as a trimer of identical subunits. Each subunit consists of two transmembrane domains connected by a large extracellular loop, with both the N‐ and the C‐termini oriented toward the cytoplasm [12]. These receptors are expressed in a wide range of excitable and non‐excitable cell types, where they contribute to the regulation of membrane excitability, intracellular calcium dynamics [13, 14], and the release of neurotransmitters [15, 16, 17] and hormones [17], as well as to the processing of pain signals [18, 19, 20, 21]. Beyond neural pain pathways, P2RX4 localizes to lysosomal compartments [22] as well as to the plasma membrane [23], enabling roles in receptor trafficking [24] or immune responses [25, 26]. P2RX4 has been observed to be upregulated in prostate cancer [27, 28], sarcoma [29], and clear cell carcinoma [30], where it conferred pro‐tumorigenic functions, including effects on tumor cell invasion, proliferation, mitochondrial metabolism [26], neo‐angiogenesis, and tumor‐associated macrophage activity. In contrast, the relative expression ratio of P2RY2 and P2RX4 in gastric cancer cells determined whether extracellular ATP promoted proliferation [31]. Thus, P2RX4 has emerged as a multifaceted contributor to tumor aggressiveness, microenvironment modulation, and metabolic adaptation.

Here, we aimed to assess P2RX4 expression in CRC and PDAC to determine whether P2RX4 can serve as a stratification marker for identifying patients who may benefit from combination therapy involving P2RX4 inhibition during chemotherapy.

## Materials and methods

### Ethical approval and patient consent

Human colorectal and pancreatic ductal adenocarcinoma organoids were collected in collaboration with the Interdisciplinary Biobank and Database Frankfurt (IBDF) and the University Cancer Center (UCT) after prior written informed consent. This study was approved by the institutional review board of UCT and the Ethics Committee at the University Hospital Frankfurt (Ethics vote: 4/09, 2022‐967, and 274/18; project numbers: SGI‐06‐2015, SGI‐12‐2018, SGI‐7‐2018, and UCT‐10‐2023).

Human tumor specimens for immunohistochemical analysis and clinical data were provided by the Senckenberg Biobank of the Dr. Senckenberg Institutes of Pathology and Human Genetics at University Hospital Frankfurt in collaboration with UCT. Written informed consent was obtained from all patients, and the study was approved by the institutional review board of the UCT and the Ethics Committee at the University Hospital Frankfurt (Ethics vote: 4/09, 274/18; project numbers: SGI‐11‐2018, SGI‐13‐2018, and UCT‐10‐2023).

### Patient‐derived colorectal cancer organoid lines

The patient‐derived colorectal cancer organoid lines O1–O4 were established from patients with the following characteristics: hCRC line O1 was derived from right‐sided adenocarcinoma tissue from a 62‐year‐old female; hCRC line O2 was established from sigmoid adenocarcinoma tissue from a 38‐year‐old female; hCRC line O3 was established from descending colon adenocarcinoma tissue from a 76‐year‐old male; and hCRC line O4 was established from sigmoid adenocarcinoma tissue from a 46‐year‐old male.

### P2X4 immunohistochemistry

Tumor tissue was reviewed by a pathologist and tissue staining was conducted using the Dako Omnis platform (Agilent, Santa Clara, CA, USA) using anti‐P2RX4 antibody (PA5‐93144; Thermo Fisher Scientific, Darmstadt, Germany; dilution 1:100, incubation time 30 min). Slides were pretreated using the EnVision FLEX low pH (Agilent) protocol; details are available upon request from the corresponding author. Staining was evaluated using a four‐tier scale (absent – score 0; weak – score 1; moderate – score 2; strong – score 3). Tumor tissue was partially evaluated using a tissue microarray (TMA) approach. For quantification, the cases were classified as follows: no P2RX4 expression (score 0), P2RX4‐low expression (scores 0.5–1.5), and P2RX4‐high expression (scores 2–3).

### Analysis of publicly available human colorectal and pancreatic cancer datasets

The following publicly available datasets were accessed and FASTQ files were downloaded and further processed as indicated.

**Figure 1:**
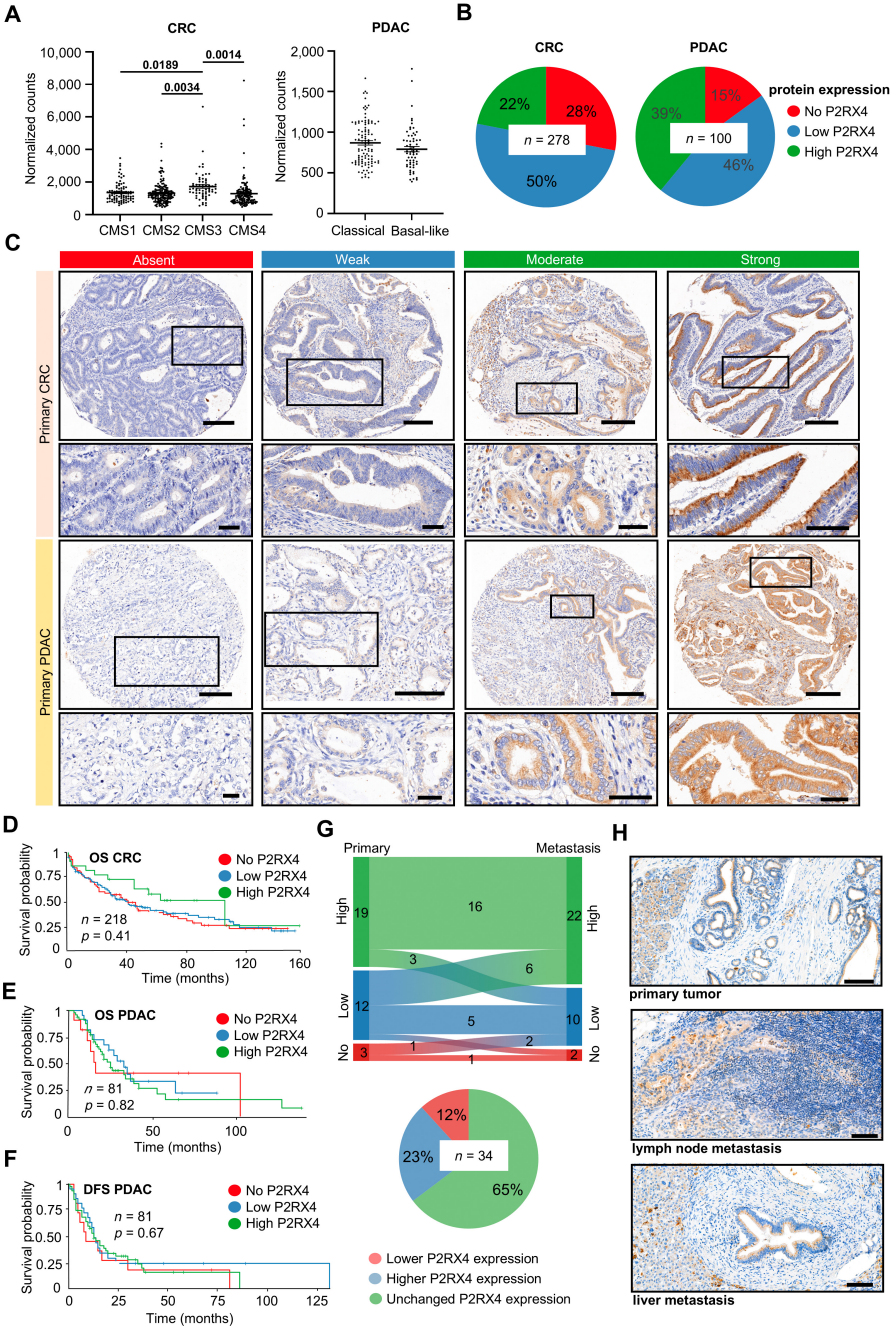
P2RX4 expression in human colorectal and pancreatic cancer. (A) *P2RX4* gene expression in primary human cancers from TCGA cohorts: COAD (colon adenocarcinoma, CRC, *n* = 592) and PAAD (pancreatic adenocarcinoma, PDAC, *n* = 177). Normalized counts are shown in FPKM (Fragments Per Kilobase of transcript per Million mapped reads). The COAD cohort is grouped according to transcriptomic consensus molecular subtypes (CMSs). The PAAD cohort is classified as classical PDAC or basal‐like subtype. Data are shown as mean ± SEM and were analyzed by a two‐tailed Student's *t*‐test (PDAC) or one‐way analysis of variance (CRC). Bold font is used for statistically significant *p* values. (B) P2RX4 protein expression in colorectal cancer (left) and pancreatic cancer (right) tissues. Immunohistochemistry‐based expression levels in tissue microarrays were categorized as no expression (red), low expression (blue), or high expression (green), with corresponding percentages indicated for each category. (C) Representative images of immunohistochemical analysis of P2RX4 in human colorectal (upper panels) and pancreatic cancer (lower panels) tissues from primary tumors. High‐magnification insets of the highlighted regions provide detailed views of staining patterns. Scale bars, 200 μm (first and third rows) and 50 μm (second and fourth rows). These images correspond to the quantified data presented in panel B. (D) Kaplan–Meier overall survival plots of 218 primary colorectal cancer patients with available clinical data, stratified by P2RX4 protein expression as shown in panel B. (E) Overall survival (OS) and (F) disease‐free survival (DFS) analysis of 81 primary pancreatic ductal adenocarcinoma patients with available survival data from panel B (right). (G) Alluvial diagram (top) and pie chart (bottom) of 34 primary PDAC tumor samples with available matched lymph node or distant metastasis, classified according to P2RX4 protein expression as in panels B and C. (H) Representative images of immunohistochemical analysis of P2RX4 in matched primary PDAC with synchronous lymph node metastasis and liver metastasis. Scale bars, 100 μm.

1. The results shown in Figure 1A are based on data generated by the TCGA Research Network (https://www.cancer.gov/tcga). The COAD and PAAD datasets used are part of the TCGA PanCancer Atlas dataset [32]. Analysis was performed by grouping CRC samples according to consensus molecular subtypes (CMSs) using the CMScaller R package [33]. PDAC samples were classified using the pdacR platform (GitHub repository: https://github.com/rmoffitt/pdacR; last accessed 23 April 2026) into classical and basal‐like subtypes [34]. Gene‐level counts were normalized using the DESeq2 R package (GitHub repository: https://github.com/thelovelab/DESeq2; last accessed 23 April 2026, version 1.44.0)
2. Data from our in‐house patient‐derived colorectal cancer organoid biobank have been previously published [35]. The CRC organoid sequencing data were previously deposited at the European Genome‐phenome Archive (https://ega-archive.org/) under accession numbers EGAS00001007300 (RNA sequencing data) and EGAS00001007301 (exome sequencing data). For gene expression analysis, the sequences were aligned to reference genome Homo sapiens (Ensembl version 111) using the STAR aligner (GitHub repository: https://github.com/alexdobin/STAR; last accessed 23 April 2026, version 2.7.11b). Gene‐level counts were summarized using HTSeq‐count (GitHub repository: https://github.com/htseq/htseq; last accessed 23 April 2026, version 2.0.5), and the counts were normalized using the DESeq2 R package (version 1.44.0). Significantly differentially expressed genes [adjusted *p* value < 0.05 and abs(log2FC) > 2] were filtered and designated as candidate genes, which were used for volcano plots and GSEA (gene set enrichment analysis) dot plots.

For transcript quantification, Salmon [36] (GitHub repository: https://github.com/COMBINE-lab/salmon; last accessed 23 April 2026, v1.10.3) was used for mapping and read counting. Ensembl annotations [37] (GRCh38 Release 111) or RefSeq [38] (NCBI Homo sapiens Annotation Release 110) was used as the reference database. Normalization was performed using DEXSeq [39].

Variant allele frequency was matched to RNA sequencing results, and known colorectal cancer‐associated genes were manually selected for molecular characterization.

3 The pancreatic cancer epithelial cell compartment was analyzed using the publicly available GSE93326 RNA sequencing dataset [40, 41, 42]. In brief, epithelial and stromal compartments were dissected as previously described [42]. Cryosections of OCT‐embedded tissue blocks were mounted on PEN membrane glass slides and stained with cresyl violet acetate. Adjacent sections were H&E‐stained for pathology review. Laser capture microdissection was performed using a PALM MicroBeam microscope (Carl Zeiss, Oberkochen, Germany), collecting at least 1,000 cells per compartment. RNA was extracted and libraries were prepared using the Ovation RNA‐Seq System V2 kit (NuGen, Toronto, Ontario, Canada). Libraries were sequenced to a depth of 30 million, 100 bp, single end reads.

Transcript quantification was performed using Salmon [36] (v1.10.3) for mapping and read counting. Ensembl annotations [37] (GRCh38 Release 111) or RefSeq [38] (NCBI Homo sapiens Annotation Release 110) was used as the reference database. Normalization was performed using DEXSeq [39].

### Survival analysis

Clinical data were provided by the University Cancer Center Frankfurt (UCT). Patients were grouped according to their immunohistochemical P2RX4 expression into three groups: no P2RX4 expression (score 0), P2RX4‐low expression (scores 0.5–1.5), and P2RX4‐high expression (scores 2–3). Progression‐free and overall survival analysis was performed using the survminer: Drawing Survival Curves using ‘ggplot2’ R package version 0.5.0 in R Studio (R software, version 4.4.3; R Foundation for Statistical Computing, Vienna, Austria) [43].

### Whole‐mount human organoid immunofluorescence

Human organoids were collected in ice‐cold medium, gently washed by sedimentation, and resuspended in 2% paraformaldehyde in phosphate‐buffered saline (PBS). Fixation was performed at 4 °C overnight. After 20‐min permeabilization in PBS with 0.1% Tween 20 (Sigma‐Aldrich, Burlington, MA, USA; #P1379) and 0.1% Triton X‐100 (Sigma‐Aldrich, #93443), cells were blocked in PBS with 0.1% Tween 20% and 2% donkey serum for 1 h at room temperature. Antibodies were applied in PBS with 0.1% Tween 20% and 2% goat serum in the following dilutions: anti‐P2RX4 antibody (Thermo Fisher Scientific, Waltham, MA, USA; #PA5‐93144) diluted 1:100 and EpCAM (Cell Signaling Technology, Danvers, MA, USA; #2929S) diluted 1:500. After washing, organoids were incubated in suitable secondary antibodies [Goat anti‐Mouse IgG (H+L) Antibody, Alexa Fluor Plus 647 (Thermo Fisher Scientific, #A32728) and Goat anti‐Rabbit IgG (H+L) Antibody, Alexa Fluor 488 (Thermo Fisher Scientific, #A‐11034), both diluted 1:1,000] for 1 h at room temperature, washed again, and slides were mounted with ProLong Gold Antifade Mountant with the DNA stain DAPI (Thermo Fisher Scientific, #P36931).

### Image capture and analysis by confocal laser scanning microscope method

Fluorescence images of *P2RX4*–SNAP‐tag constructs and hCRC organoids were acquired using a Leica TCS SP5 confocal laser scanning microscope (Leica Microsystems CMS GmbH, Mannheim, Germany) with LAS AF (Leica Application Suite Advanced Fluorescence) software (version 1.8.1, build 1390). Imaging was performed using an HCX PL APO lambda blue oil‐immersion objective (63.0×, NA 1.40). The confocal pinhole was set to 2 Airy units, and bidirectional scanning was applied. An optical zoom factor of 4–6× was used. Images were acquired at 12‐bit depth. Further details on image analysis and processing are provided in the Supplementary materials and methods.

### Statistical analyses

Calculation of statistical significance was done using GraphPad Prism 10 (GraphPad Software Inc., San Diego, CA, USA) applying one‐way ANOVA, as well as an unpaired or a paired *t*‐test. The data are presented as mean ± SD; the number of samples (*n*) is indicated in the graph or in the figure legends. Statistical significance is indicated as follows: *p < 0.05, **p < 0.01, ***p < 0.001 and ****p < 0.0001. Error bars correspond to 95% confidence interval and were plotted by GraphPad Prism (version 10). All measurements were taken from distinct samples; no samples were measured repeatedly to generate data.

## Results

### P2RX4 expression in human CRC and PDAC

To investigate whether putative changes in *P2RX4* gene expression allow patient stratification, we analyzed bulk RNA sequencing data of CRC and PDAC patients from the publicly available database ‘The Cancer Genome Atlas Program’ (TCGA). *P2RX4* gene expression was detected in all specimens across both cancer types (Figure 1A). While colorectal consensus molecular subtype 3 (CMS3) tumors showed a small yet statistically significant higher overall expression, we did not find any marked differences among the other subtypes. Furthermore, between classical and basal‐like subtypes [34] of PDAC, we did not observe a difference in *P2RX4* gene expression (Figure 1A). However, at the protein level, we observed substantial alterations and more heterogeneous expression between patients when performing immunohistochemical analysis of P2RX4 in tissue microarrays consisting of primary CRC (*n* = 278) and PDAC (*n* = 100) samples (Figure 1B). Among these, 22% of CRC samples and 39% of PDAC samples exhibited high P2RX4 protein expression (Figure 1B) characterized by a granular apical staining pattern in cancer cells but not in stromal compartments (Figure 1C). Samples with low P2RX4 expression displayed a less granular and more diffuse staining pattern (Figure 1C). In contrast to gene expression, the P2RX4 protein was entirely absent in 28% of CRC samples and 15% of PDAC samples (Figure 1B). Interestingly, differences in P2RX4 expression were not coupled to changes in overall survival for CRC patients (Figure 1D) and also had no impact on overall or progression‐free survival of PDAC patients (Figure 1E,F), in agreement with previous observations that *P2RX4* gene expression did not correlate with survival [44]. To examine whether P2RX4 expression in metastases correlated with expression in primary tumors, an additional PDAC cohort consisting of 34 primary tumor specimens with matched synchronous lymph node and/or distant metastases was analyzed. In two‐thirds of the patients (65%), P2RX4 expression levels were comparable between primary tumors and metastases (Figure 1G,H). In 23% of PDAC patients, we found higher P2RX4 expression in metastases, while 12% of patients (*n* = 4 out of 34) showed a stronger staining pattern in primary tumors (Figure 1G). Collectively, our findings revealed a marked discrepancy between *P2RX4* gene and protein expression in human PDAC and CRC. However, P2RX4 expression levels did not correlate with clinical outcomes, while intra‐individual expression patterns between primary tumors and corresponding metastases showed a high degree of concordance.

### P2RX4 is alternatively spliced in human colorectal organoids and pancreatic cancer epithelial cells

**Figure 2:**
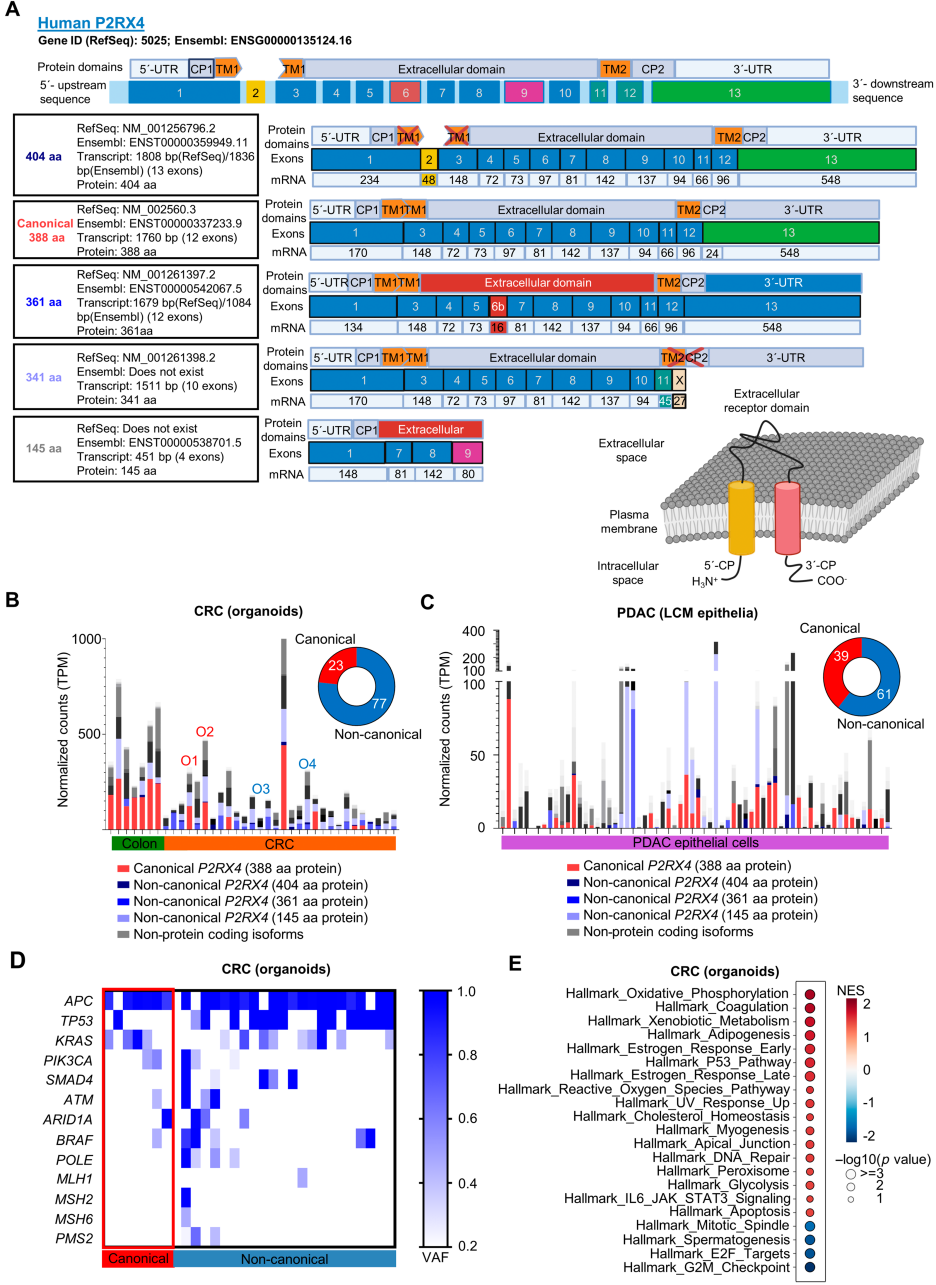
Human *P2RX4* is alternatively spliced in colorectal and pancreatic cancer. (A) Schematic representation of the human *P2RX4* gene, its protein‐coding transcripts, and functional protein domains. Matching NCBI RefSeq and Ensembl IDs are provided, along with additional overview data on the left. Exon lengths (in base pairs) are displayed for each exon, and the corresponding protein domains are mapped to their respective exons. The labels on the left are based on the corresponding protein length in amino acids (red color marking the canonical P2RX4 isoform and blue colors the non‐canonical isoforms). Alternative exons and the proposed resulting changes in the protein sequence are indicated for each variant. Additionally, a schematic of the canonical P2RX4 protein and its functional protein domains is displayed (lower right corner). Created in BioRender. Steup, C. (2026) https://BioRender.com/tihymnx. (B) Bulk RNA sequencing‐based *P2RX4* transcript expression (Reference Database: Ensembl 111) in human colon and colorectal cancer organoids. Each bar represents one patient‐derived (tumor) organoid. Grey color shades indicate non‐protein‐coding *P2RX4* transcripts. The pie chart indicates the percentage of samples with the canonical *P2RX4* transcript (red) and non‐canonical protein‐coding transcripts (blue). (C) Analysis of bulk RNA sequencing data [42] for *P2RX4* transcript expression in tumor cells isolated by laser captured microdissection (LCM) from fresh frozen primary human pancreatic adenocarcinoma. Each bar represents one patient sample. The pie chart shows the percentage of samples with the canonical *P2RX4* transcript (red), non‐canonical protein‐coding transcripts (blue), and non‐protein‐coding transcripts (grey). (D) Variant allele frequency (VAF) in selected frequently mutated genes from human colorectal cancer organoids. Organoids expressing the canonical (*n* = 7) or non‐canonical (*n* = 23) *P2RX4* transcripts are indicated. (E) Hallmark gene set enrichment analysis [66] comparing canonical human CRC organoids (from panel D) with non‐canonical organoids.

Both full‐length and alternatively spliced *P2RX4* mRNAs are expressed across multiple tissues, yet only the full‐length transcript encodes a functional ion channel [45]. The canonical P2RX4 protein consists of a C‐ and an N‐terminal cytoplasmic domain, two transmembrane domains, and a long extracellular receptor domain. The proper formation of the first transmembrane domain (TM1) requires splicing and deletion of exon 2 (Figure 2A). To examine whether alternative splicing of *P2RX4* could account for the differences observed between transcriptomic data and the IHC results, we utilized our patient‐derived tumor organoid (PDTO) biobank [35]. Intriguingly, using the Ensembl genome browser (annotation 111) as a reference database, we observed the presence of various non‐canonical protein‐coding *P2RX4* transcripts in the majority of colorectal PDTOs (77%; Figure 2B). These comprised a 404‐amino acid (aa) isoform generated by retention of exon 2, resulting in disruption of TM1; a 361‐aa isoform arising from an exon 6 deletion, thereby modifying the extracellular domain; and a 145‐aa isoform lacking both transmembrane domains and exhibiting a truncated extracellular domain (Figure 2A). In addition, we detected a relevant proportion of *P2RX4* transcripts without protein‐coding function (Figure 2B). The frequent presence of non‐canonical isoforms could also be confirmed using an alternative database (NCBI RefSeq database; annotation release 110) (supplementary material, Figure S1A,B). However, instead of the 145‐aa isoform defined by the Ensembl genome browser, the NCBI RefSeq database annotated a different transcript encoding a 341‐aa isoform that is characterized by a C‐terminal truncation, thus disrupting TM2 and the C‐terminal cytoplasmic domain (Figure 2A). Nevertheless, the overall ratio of canonical to non‐canonical isoforms was comparable between the two databases. Unfortunately, in bulk tumor samples, we were unable to resolve this pattern, as background sequencing noise from surrounding non‐epithelial cells expressing relevant levels of the canonical *P2RX4* transcript obscured isoform‐specific signals (data not shown). Therefore, we analyzed tumor cell transcriptomes obtained from 65 laser capture‐microdissected PDACs [40, 41, 42]. As in CRC organoids, non‐canonical *P2RX4* isoforms (404 aa, 361 aa, and 145 aa) were more frequent than canonical transcripts in pancreatic tumor cells, although the incidence of the latter was higher (39%; Figure 2C) than in CRC PDTOs (23%; Figure 2B).

To explore potential links between different *P2RX4* isoforms and key CRC driver mutations, we analyzed our CRC biobank containing 30 molecularly well‐characterized tumor organoids [35]. Among these, seven predominantly expressed the canonical *P2RX4* isoform (388 aa; exon 2 deletion), whereas 23 primarily expressed non‐canonical *P2RX4* transcripts (Figure 2B). Due to this uneven distribution, definitive correlations could not be established. Nevertheless, we observed a trend suggesting a higher frequency of *TP53* mutations, and to a lesser extent *SMAD4* mutations, in the non‐canonical group (Figure 2D). At the transcriptomic level in CRC organoids, we identified 432 genes upregulated and 289 genes downregulated in the canonical group compared with the non‐canonical group (supplementary material, Figure S1C). Gene set enrichment analysis (GSEA) of hallmark pathways revealed upregulation of p53 signaling, reactive oxygen species, DNA repair, and IL6–JAK–STAT3 pathways, while G2M checkpoint and mitotic spindle pathways were significantly downregulated in canonical CRC organoids (Figure 2E).

### Isoform‐specific P2X4–SNAP‐tag fusion proteins differ in localization and abundance

**Figure 3:**
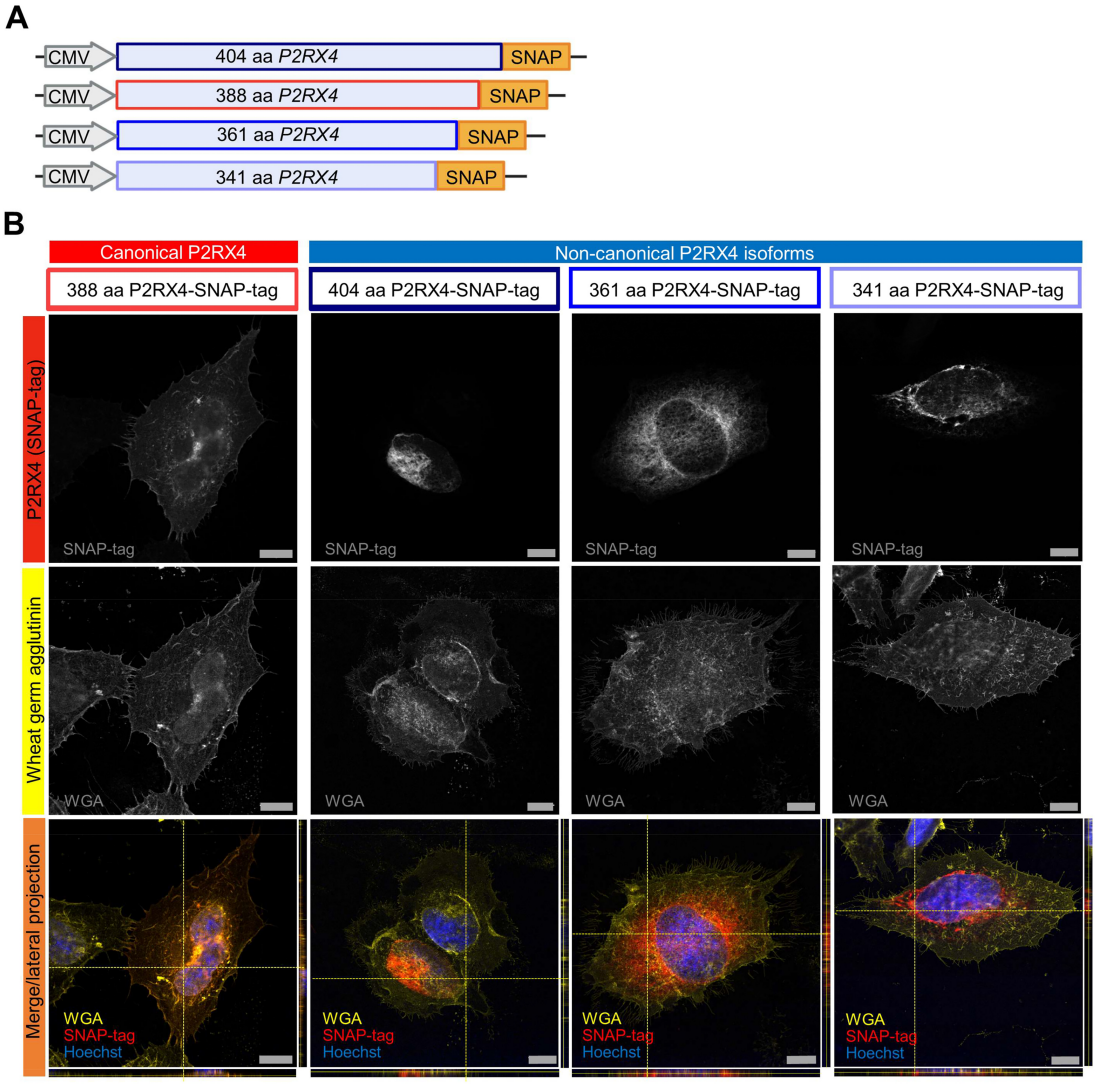
Membrane localization of human P2RX4–SNAP‐tag fusion constructs. (A) Schematic illustration of *P2RX4*–SNAP‐tag expressing vectors. A 20 kDa SNAP‐tag [67] was added to the C‐terminus of the relevant protein‐coding *P2RX4* transcripts shown in Figure 2A. Protein‐coding P2RX4 isoforms are labeled by their protein lengths. For the full vector map and sequences, refer to the Supplementary materials and methods section. (B) Representative confocal microscopy images. HeLa cells were transfected with *P2RX4*–SNAP‐tag constructs and labelled after 1 day. The cells were fixed and counterstained with wheat germ agglutinin (WGA; membrane marker) and Hoechst (nuclear marker). Scale bars, 10 μm. In the merged images, lateral projections from the indicated yellow lines of 0.5 μm thick *z*‐stacks are shown. The Hoechst channel is displayed only in the merged images.

**Figure 4:**
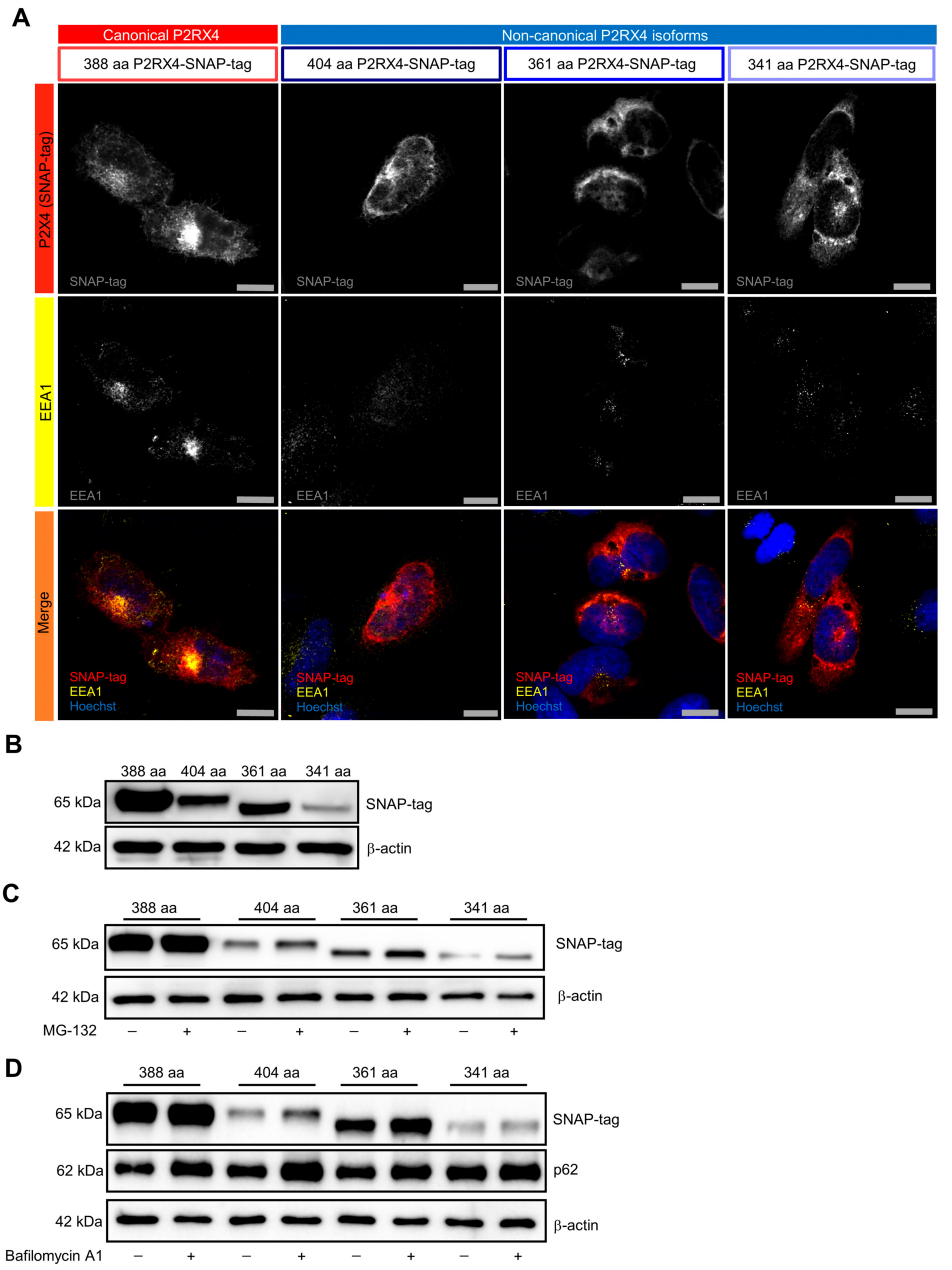
Protein localization and abundance of P2RX4–SNAP‐tag fusion constructs. (A) Representative confocal microscopy images of HeLa cells transfected with the respective *P2RX4*–SNAP‐tag constructs and labelled after 1 day with TMR‐Star reagent for SNAP‐tag constructs. The cells were counterstained with EEA1 (early endosomal marker) and Hoechst. Scale bars, 10 μm. The Hoechst channel is displayed only in the merged images. (B) Representative immunoblots of HEK293T cells transfected with equal amounts of *P2RX4*–SNAP‐tag constructs. Immunoblotting analysis was performed 24 h post‐transfection (*n* = 3). (C) Representative immunoblotting of HEK293T cells transfected with equal amounts of *P2RX4*–SNAP‐tag constructs and treated 18 h post‐transfection for 6 h with MG‐132 (*n* = 3). (D) Representative immunoblotting of HEK293T cells transfected with equal amounts of *P2RX4*–SNAP‐tag constructs. The treatment with Bafilomycin A1 was initiated 6 h post‐transfection and lasted for 18 h (*n* = 3).

To examine whether the different functional protein domains encoded by the P2RX4 isoforms would impact P2RX4 localization and abundance, we generated fusion constructs of the respective protein‐coding *P2RX4* transcripts with a C‐terminal 20 kDa SNAP‐tag [46] (Figure 3A). Due to multiple premature stop codons in the 145‐aa isoform (supplementary material, Figure S2), we refrained from generating an additional 145‐aa SNAP‐tag fusion construct. We expressed the different variants in HeLa cells and visualized the SNAP‐tagged isoforms and the plasma membrane by immunofluorescence (Figure 3B; supplementary material, Videos S1–S4). Only canonical P2RX4–SNAP‐tag protein could be detected at the plasma membrane, whereas none of the three non‐canonical P2RX4–SNAP‐tag isoforms (404 aa, 361 aa, 341 aa) were observed (Figure 3B; supplementary material, Videos S1–S4). Rather, these non‐canonical variants retained cytoplasmic expression and co‐localized with the endoplasmic reticulum (supplementary material, Figure S3A), lysosomes (supplementary material, Figure S3B), and mitochondria (supplementary material, Figure S4A), indicating that aberrant protein formation impaired their transport to the cell membrane. Furthermore, unlike canonical P2RX4, which was clearly detected in EEA1‐positive early endosomes (Figure 4A), the non‐canonical isoforms were absent from these compartments, indicating a lack of P2RX4 endocytosis. We next performed immunoblotting analysis using an antibody that specifically recognizes the P2RX4*–*SNAP‐tag protein without any binding to endogenous P2RX4 protein. In line with the frequent downregulation of P2RX4 protein associated with the non‐canonical P2RX4 isoforms in human CRC and PDAC, protein expression of the non‐canonical P2RX4 fusion constructs was markedly lower than that of the 388‐aa canonical isoform (Figure 4B). Since expression levels of the *P2RX4*–SNAP‐tag constructs were comparable (data not shown), we tested whether inhibition of protein degradation would affect the abundance of P2RX4–SNAP‐tag transcript variants. Indeed, treatment with the proteasome inhibitor MG‐132 (Figure 4C) or inhibition of autophagy using bafilomycin A1 (Figure 4D) increased the protein levels of non‐canonical P2RX4–SNAP‐tag variants but not those of the canonical, indicating that aberrant P2RX4 isoforms are routinely eliminated by proteasomal degradation and autophagy.

### Canonical *P2RX4* expression predicts therapy response to P2RX4 inhibition in combination with chemotherapy

**Figure 5:**
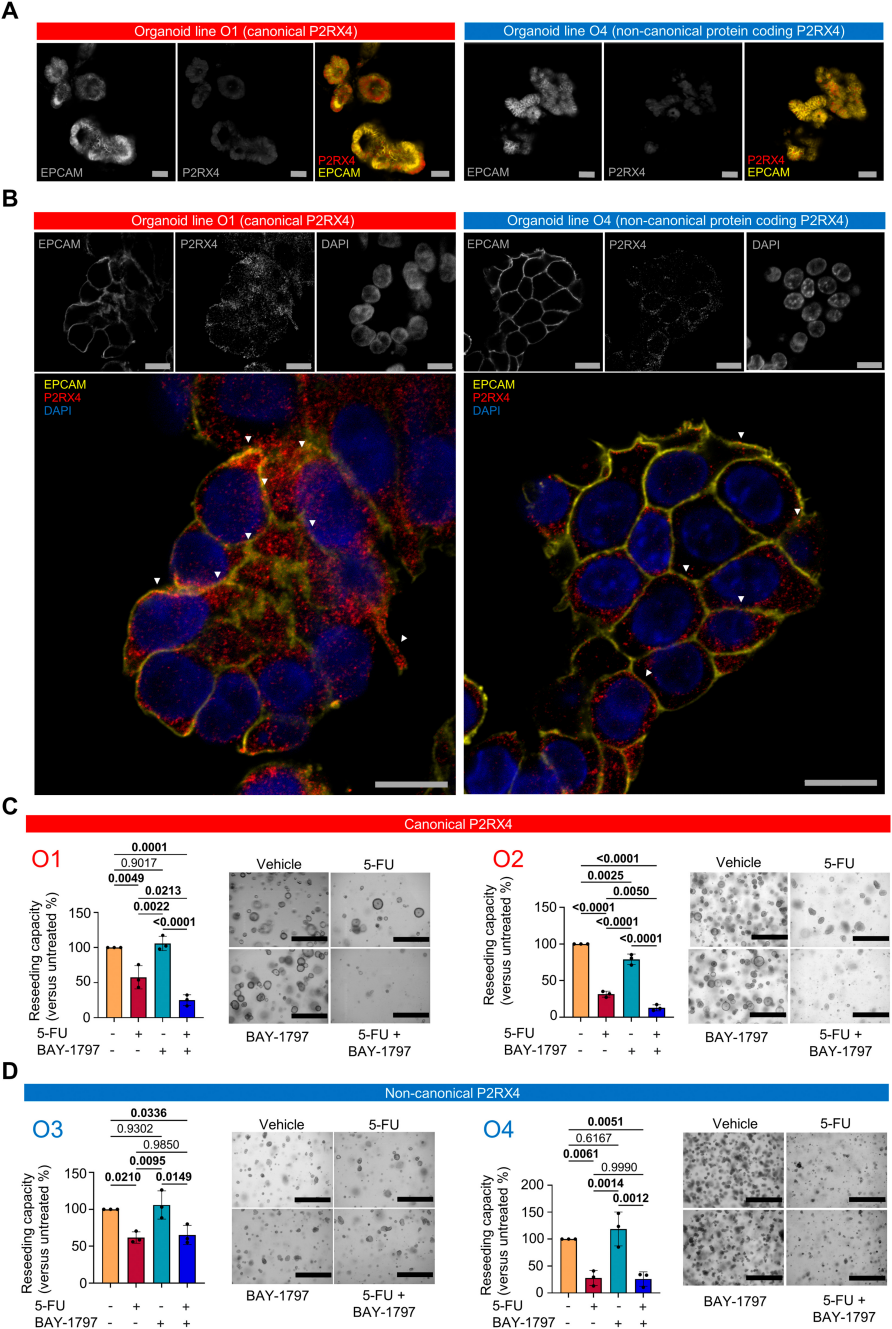
P2RX4 expression in human colorectal and pancreatic cancer organoids. (A, B) Human colorectal cancer organoids expressing the canonical *P2RX4* transcript (O1, left) or exclusively expressing non‐canonical *P2RX4* transcripts (O4, right) were selected for imaging. (A) Representative light sheet immunofluorescence microscopy images stained for P2RX4 and EPCAM. Scale bars, 50 μm. (B) Representative confocal microscopy images stained for P2RX4 and EPCAM and counterstained with DAPI. Scale bars, 10 μm. (C) Reseeding capacity of human colorectal cancer organoid lines O1 and O2 expressing the canonical *P2RX4* transcript treated as indicated (*n* = 3). Representative images were taken 14 days after reseeding. Scale bars, 1,000 μm. All data are shown as mean ± SD and were analyzed by one‐way analysis of variance (ANOVA). Bold font is used for statistically significant *p* values. (D) Reseeding capacity of human colorectal cancer organoid lines O3 (without relevant expression of protein‐coding *P2RX4*) and O4 (expressing protein‐coding non‐canonical *P2RX4*) treated as indicated (*n* = 3). Representative images were taken 14 days after reseeding. Scale bars, 1,000 μm. All data are shown as mean ± SD and were analyzed by one‐way analysis of variance (ANOVA). Bold font is used for statistically significant *p* values.

To assess whether detection of canonical *P2RX4* could be used to stratify cancer patients who might benefit from additional P2RX4 inhibition during standard‐of‐care chemotherapy, we analyzed PDTOs from our CRC biobank. For that purpose, we picked PDTOs analyzed by RNA sequencing (Figure 2B) that either expressed the canonical *P2RX4* isoform (lines O1 and O2) or the non‐canonical transcripts (lines O4 protein‐coding and non‐protein‐coding non‐canonical transcripts) and O3 (exclusively non‐protein‐coding non‐canonical transcripts as a negative control). Consistent with the results from SNAP‐tag fusion proteins, 3D visualization using light sheet microscopy revealed membranous P2RX4 expression in the canonical line O1 (Figure 5A; supplementary material, Video S5), but not in the non‐canonical line O4 (Figure 5A; supplementary material, Video S6). As the resolution was insufficient to draw final conclusions regarding P2RX4 localization at the plasma membrane, we performed confocal microscopy of the same organoid lines. As expected, overall P2RX4 staining signal was stronger and co‐localization with the EPCAM‐positive plasma membrane was higher in the canonical lines O1 and O2 than in the non‐canonical lines O3 and O4 (Figure 5B; supplementary material, Figure S5A). The non‐canonical organoid line O4 (Figure 5B) showed a P2RX4 expression pattern in the perinuclear area similar to that observed with our non‐canonical P2RX4–SNAP‐tag isoforms (Figure 3B). The non‐canonical organoid line O3, which lacks a relevant protein‐coding P2RX4 transcript, showed no detectable P2RX4 staining (supplementary material, Figure S5A). Finally, we examined whether canonical P2RX4 expression was required for therapy response. We treated both the canonical and the non‐canonical lines with either 5‐fluorouracil (5‐FU), a highly selective P2RX4 inhibitor (BAY‐1797), or a combination of both. BAY‐1797 monotherapy did not impact organoid survival in any of the lines (Figure 5C,D). However, in the presence of 5‐FU, BAY‐1797 significantly enhanced organoid death and suppressed the reseeding capacity of PDTOs in the canonical lines expressing membranous P2RX4 addition (Figure 5C), which was not observed in the non‐canonical lines (Figure 5D). Collectively, these data confirm that only the canonical isoform enables membranous P2RX4 expression, which is a prerequisite for effective modulation of paracrine cell death‐associated P2RX4 engagement.

## Discussion

Non‐genetic transient chemotherapy resistance induced by dying cancer cells in the tumor microenvironment is an important mechanism of treatment resistance in solid cancer [47]. Recently, we suggested that targeting P2RX4 engaged signaling may represent a promising strategy to overcome such resistance [11]. Assessing whether differences in P2RX4 expression could help to stratify patients who might benefit from P2RX4 inhibition during chemotherapy, we surprisingly found no marked changes in gene expression but instead pronounced variability in the protein levels among both colorectal and pancreatic cancer patients. Moreover, protein expression between primary tumors and metastatic lesions indicated a high level of concordance within individual patients, suggesting that immunohistochemical analysis of a single biopsy of either primary or metastatic sites might suffice for pre‐treatment stratification.

Alternative splicing of *P2RX4* has been previously described in neurons [45], and we now demonstrate that altered splicing is responsible for the downregulation and loss of the canonical *P2RX4* transcript and the appearance of non‐canonical *P2RX4* transcripts in colorectal and pancreatic tumor epithelia. Alternative pre‐mRNA splicing [48] is a central mechanism that increases proteomic complexity by generating multiple transcripts from a single gene through spliceosome‐mediated RNA‐processing in most human multi‐exon genes [49]. Apart from its physiological role in, for example, development [49], tissue differentiation [49, 50], and T‐cell activation [51, 52], large‐scale transcriptomic analyses have revealed widespread dysregulation of RNA splicing across most tumor types, with tumors exhibiting substantially more alternative splicing events compared with peritumoral healthy tissue [53]. Tumor‐specific alterations in splicing occur through *cis*‐acting somatic mutations or altered expression of *trans*‐acting splicing factors, and any such alterations in splicing patterns have the potential to drive pro‐tumorigenic alterations of cancer [54, 55]. Typically, mutations in genes encoding for splicing factors occur early in tumorigenesis [56, 57, 58, 59, 60, 61], highlighting their importance for cancer development and progression. In line with this, we detected alterations in *P2RX4* transcript abundance already in localized human CRC and PDAC. Despite the pronounced alterations in P2RX4 splicing, particularly in CRC, overall survival was not correlated with P2RX4 protein levels. Therefore, P2RX4 regulation appears to be a bystander event downstream of general spliceosome alteration during CRC and PDAC tumorigenesis rather than conferring a selective advantage. In addition, we observed that P2RX4 protein expression in both CRC and PDAC is restricted to cancer cells, sparing most of the stromal compartments. One possible explanation is that tissue‐ and cell‐specific regulation of canonical P2RX4 expression arises from epithelial differentiation programs in the colon and pancreas and is lost upon dedifferentiation during cancer formation. Consistent with this, tissue‐specific RNA‐sequencing data from GeneCards indicate relatively uniform *P2RX4* gene expression across healthy tissues, but a wide range of P2RX4 protein expression levels [62]. Post‐translational modification of canonical (full‐length) P2RX4 is required for its trafficking to the plasma membrane [63]. Accordingly, defective post‐translational modification resulting from changes in the protein sequence and/or localization may contribute to impaired membrane localization and increased degradation, particularly for the 361‐aa P2RX4 isoform, which retains intact transmembrane domains. Nevertheless, P2RX4 abundance within the lysosomal compartment appears unaffected, possibly due to the repetitive lysosomal targeting through a dileucine‐type motif within the N‐terminus [22] and a tyrosine‐based motif in the C‐terminus [64]. Moreover, misfolded proteins are degraded within lysosomes downstream of autophagic pathways. Therefore, although overall lysosomal co‐localization remained unchanged in our model, the trafficking route to lysosomes may differ.

We did not identify any relevant mutations of known splicing factors, nor did we identify a clear correlation of P2RX4 expression with the frequency of any CRC driver mutations. Thus, we expect that a combination of genetic alterations, likely in a tissue‐specific manner, is leading to mis‐interaction or mis‐localization of one or more of the approximately 300 nucleic acids and proteins involved in the spliceosome complex. Unfortunately, further evaluation in a murine *in vivo* model system, to genetically and mechanistically dissect the different oncogenic mutations and extracellular signaling cues on *P2RX4* splicing, is limited by interspecies differences in splicing isoforms. In particular, murine P2RX4 protein isoforms differ predominantly in the extracellular receptor domain rather than in the transmembrane domain.

Collectively, we identify aberrant splicing of *P2RX4* transcripts as an important regulator of P2RX4 protein expression in PDAC and CRC, possibly rendering a large fraction of patients resistant to P2RX4‐targeted treatment approaches. Therefore, we propose that future clinical trials exploring the promising approach of combining chemotherapy and P2RX4 blockade to overcome cell death‐induced chemotherapy resistance should focus on P2RX4‐high expressing tumors based on immunohistochemical analysis from a single patient biopsy. Moreover, given the high percentage of P2RX4‐high expression tumors and low inter‐lesion heterogeneity, together with the high and unmet medical need for therapies targeting non‐genetic chemotherapy resistance [65], we propose that PDAC represents a promising entity for future P2RX4‐targeted clinical trials.

## Author contributions statement

FRG and PKZ were responsible for conceptualization. FRG, CS, PW and PKZ conducted the methodology. CS, JD, CD‐F, SK, EE, AFdV, KBK, DM, MK and PKZ carried out investigations. PJW, FRG and PZ provided resources. CS, PKZ and FRG wrote the original draft. CS, CD‐F and FRG performed visualization. PKZ and FRG were responsible for supervision and funding acquisition. All authors approved the final version of the manuscript.

## Supporting information


Supplementary materials and methods
**Figure S1**. Additional characterization of *P2RX4* transcript expression in human colorectal and pancreatic cancer
**Figure S2**. Protein and mRNA sequences of human P2RX4 isoforms
**Figure S3**. P2RX4–SNAP‐tag fusion constructs in the endoplasmic reticulum and lysosomal compartment
**Figure S4**. P2RX4–SNAP‐tag fusion constructs and mitochondria
**Figure S5**. P2RX4 expression in additional human colorectal cancer organoids

**Video S1.** Expression of canonical (388 aa) P2RX4–SNAP‐tag
**Video S2**. Expression of non‐canonical 404‐aa P2RX4–SNAP‐tag
**Video S3**. Expression of non‐canonical 361‐aa P2RX4–SNAP‐tag
**Video S4**. Expression of non‐canonical 341‐aa P2RX4–SNAP‐tag
**Video S5**. Light sheet fluorescence microscopy of human colorectal cancer organoid line O1
**Video S6**. Light sheet fluorescence microscopy of human colorectal cancer organoid line O4

## Data Availability

The data that support the findings of this study are available from the corresponding author upon reasonable request.
