# Supplementary material for: Aberrant alternative splicing of purinergic receptor P2RX4 prevents sensitivity towards combinatorial treatment in colorectal and pancreatic cancer
Source: J Pathol. 2026 Jun 3;270(1):55–68. doi: 10.1002/path.70082 (PMC13431944; doi:10.1002/path.70082)
Supplement: Supplementary file 1 — Supplementary materials and methods Figure S1. Additional characterization of P2RX4 transcript expression in human colorectal and pancreatic cancer Figure S2. Protein and mRNA sequences of human P2RX4 isoforms Figure S3. P2RX4–SNAP‐tag fusion constructs in the endoplasmic reticulum and lysosomal compartment Figure S4. P2RX4–SNAP‐tag fusion constructs and mitochondria Figure S5. P2RX4 expression in additional human colorectal cancer organoids [file PATH-270-55-s001.docx]

**Aberrant alternative splicing of purinergic receptor P2RX4 prevents sensitivity towards combinatorial treatment in colorectal and pancreatic cancer**

C Steup, *et al. J Pathol* <https://doi.org/10.1002/path.70082>

**Supplementary materials and methods**

**Supplementary Figures S1–S5**

**(Supplementary Videos S1–S6 are provided in a separate Powerpoint file)**

Reference numbers refer to the main text list

**Supplementary materials and methods**

*Cell lines*

All cell lines (HEK293T, HeLa) were originally obtained from ATCC (Manassas, VA, USA) and maintained in Dulbecco’s Modified Eagle Medium (DMEM) supplemented with 10% fetal bovine serum (FBS) and 1% penicillin–streptomycin (Pen/Strep) under standard conditions at 37 °C in a 5% CO_2_ humidified incubator. The cell lines were routinely tested for mycoplasma contamination.

*P2RX4*–SNAP-tag vectors

### The mammalian gene expression vectors used in our study were generated by adding a C-terminal SNAP-tag [45] fusion construct to the open reading frames of the human protein coding P2RX4 variants. The following vectors were constructed: pRP[Exp]-Bsd-CMV>hP2RX4[NM_002560.3]/SNAP (VB240321-1283bks), pRP[Exp]-Bsd-CMV>hP2RX4[NM_001256796.2]/SNAP ([VB240321-1259dhm](https://en.vectorbuilder.com/vector/VB240321-1259dhm.html)), pRP[Exp]-Bsd-CMV>hP2RX4[NM_001261397.2]/SNAP ([VB240826-1212njh](https://en.vectorbuilder.com/vector/VB240826-1212njh.html)), and pRP[Exp]-Bsd-CMV>hP2RX4[NM_001261398.2]/SNAP ([VB240902-1044yye](https://en.vectorbuilder.com/vector/VB240902-1044yye.html)). Constructs were validated by restriction enzyme digestion and packaged using VectorBuilder (Chicago, IL, USA; <https://en.vectorbuilder.com/>). The vector IDs in parentheses can be used to retrieve detailed information about the vector, including the full sequence. Vector maps are provided in supplementary material, Figure 3A.

*Protein isolation and immunoblotting*

For immunoblotting analysis, HEK293 cells were plated in a six-well plate. After 24 h, cells were transfected with the *P2RX4*–SNAP-tag vectors using the Lipofectamine 3000 reagent (Thermo Fisher Scientific, Darmstadt, Germany; #L3000008) following the manufacturer’s protocol. In brief, 5 µl of Lipofectamine 3000 reagent was prepared in 125 µl of Opti-MEM™ (Thermo Fisher Scientific, #31985062) and another tube was prepared with 5 µl of P3000 reagent, 2.5 µg of vector DNA in 125 µl of Opti-MEM™. Tubes were mixed and incubated for 15 min at room temperature. Vector–Lipofectamine mix (250 µl) was added dropwise to each well and the slides were gently rocked to ensure even distribution. The slides were incubated under standard conditions for 6 h and the media were changed to standard culture media or, if indicated in the figure legend, standard culture media containing 10 mm MG-132 or 1 µm Bafilomycin A1 (Cell Signaling Technology, Leiden, The Netherlands; #54645). After 24 h, cells were scraped and collected in ice-cold PBS and pelleted by centrifugation. To prepare protein lysates for immunoblotting analysis, the cell pellet was washed twice with cold PBS and the pellet lysed in lysis buffer (RIPA Buffer, Cell Signaling Technology; #CS 9806) supplemented with 0.1 mm phenylmethylsulfonyl fluoride (PMSF; Cell Signaling Technology, #8553S) on ice. Lysis was performed for 30 min, and cellular debris was removed by centrifugation.

Protein quantification was performed using the Bio-Rad Protein Assay (Bio-Rad, Bio-Rad Laboratories, Feldkirchen, Germany; #5000006) using a NanoDrop 2000c spectrophotometer (Thermo Fisher Scientific). Total protein lysate (30 μg) in Laemmli buffer (Bio-Rad Laboratories, #161-0747) was separated by SDS–polyacrylamide gel electrophoresis, transferred to a 0.45-μm PVDF membrane (Millipore, Merck, Darmstadt, Germany; #IPVH00010), and blocked with 5% non-fat milk in PBS-Tween at room temperature for 1 h. After blocking, the membranes were incubated at 4 °C overnight with primary antibodies. The following antibodies were used: SNAP-tag (New England Biolabs, Frankfurt, Germany; #P9310S; 1:1,000), SQSTM1/p62 (Abnova, Taipei City, Taiwan; #H00008878-M01; 1:1,000), and β-actin (Sigma-Aldrich, St Louis, MO, USA; #A4700; 1:1,000). After washing with PBST, membranes were incubated with appropriate horseradish peroxidase (HRP)-conjugated secondary antibodies [Mouse Anti-Rabbit IgG–HRP (Santa Cruz Biotechnology, Heidelberg, Germany; #sc-2357; 1:5,000) and Rabbit Anti-Mouse IgG H&L (HRP; Abcam, Cambridge, UK; #ab6728; 1:5,000)] for 1 h at room temperature, and membranes were developed using a SuperSignal West Pico Chemoluminescence kit (Thermo Fisher Scientific, #34580) using a chemiluminescent imager (Azure 300; Azure Biosystems, Dublin, CA, USA).

*3D immunofluorescence microscopy*

A light sheet microscope (UltraMicroscope Blaze; Miltenyi Biotec, Bergisch Gladbach, Germany) was used for 3D immunofluorescence microscopy. Permeabilization, immunostaining, dehydration, and tissue clearing of organoids were achieved by using the MACS clearing kit (Miltenyi Biotec, #130-126-719) following the manufacturer’s protocol. In brief, samples were fixed in 4% paraformaldehyde for 20 min, washed in PBS, and permeabilized in permeabilization solution for 6 h. Primary antibodies were diluted 1:100 and samples were incubated at 37 °C for 40 h under constant agitation. After washing, secondary antibody incubation was conducted under light protection as described before. After a further washing step, samples were embedded in 1% low-melting agarose and dehydrated in ethanol/H_2_O-Tween 20 solutions with increasing concentrations of ethanol. Dehydrated samples were optically cleared with the provided clearing solution and imaged at the microscope. Image processing was conducted using Imaris software (Oxford Instruments, Abingdon, UK; version 10.02).

### *Image capture and analysis by confocal laser scanning microscope method*

### For analysis of subcellular localization, HeLa cells were seeded and cultured in the four-well Nunc Lab-Tek Chamber Slide system. After 24 h, cells were transfected with the *P2RX4–SNAP*-tag vectors using the Lipofectamine 3000 reagent (Thermo Fisher Scientific, #L3000008) following the manufacturer’s protocol. In brief, 4 µl of Lipofectamine 3000 reagent was prepared in 100 µl of Opti-MEM (Thermo Fisher Scientific, #31985062) and another tube was prepared with 4 µl of P3000 reagent, 2 µg of vector DNA, and 100 µl of Opti-MEM. Tubes were mixed and incubated for 15 min at room temperature. 200 µl of vector–Lipofectamine mix was added dropwise to each well and the slides were rocked gently for distribution. The slides were incubated under standard conditions for 6 h, and the media were changed to standard culture media. One day after transfection, cells were stained using 400 µl of 3 µm SNAP-Cell TMR-Star (New England Biolabs, #S9105S) reagent in full growth medium per well for 30 min at 37 °C. SNAP-Cell TMR-Star was removed and 2 µm Hoechst 33342 solution (Thermo Fisher Scientific, #62249) was added and incubated for 5 min. Afterwards, the wells were washed three times with full growth media and finally incubated for 30 min to allow fluorophores to diffuse out of the cells. The wells were then fixed using a 4% formaldehyde solution (Sigma-Aldrich, HT501128-4L) for 10 min at room temperature. The wells were washed three times with PBS. For co-staining with Alexa Fluor 488-conjugated wheat germ agglutinin (WGA; Thermo Fisher Scientific, #W11261), cells were not permeabilized. For antibody-based co-staining, cells were permeabilized using a 0.1% Triton X-100 in PBS solution for 15 min at room temperature. For the TOM20 staining, 0.1% (w/v) saponin (Sigma-Aldrich, #STS0032-50G) was used instead during the whole staining process after 15 min pre-treatment. WGA was added with a final concentration of 5 µg/ml in HBSS and incubated under agitation for 10 min at room temperature. Afterwards, the wells were washed three times with PBS-Tween (0.05% Tween-20).

### For antibody-based co-staining, the wells were washed after permeabilization, blocked for 1 h with 1% bovine serum albumin in PBST, and primary antibody was added overnight at 4 °C in PBST. The following primary antibodies were used: calreticulin (Thermo Fisher Scientific, #MA5-15382) diluted 1:200, EEA1 (Cell Signaling Technology, #48453) diluted 1:200, TOM20 (Santa Cruz Biotechnology, #sc-17764) diluted 1:200, and LAMP2 (Abcam; #ab13524) diluted 1:200. After primary antibody incubation, slides were incubated with fluorochrome-labelled secondary antibodies for 1 h at room temperature. The following secondary antibodies were used in 1:1,000 dilution: Alexa Fluor 594-conjugated goat anti-rat IgG (Thermo Fisher Scientific, #A-11007) and Alexa Fluor 488-conjugated donkey anti-mouse IgG (Thermo Fisher Scientific, #A-21202). Slides were washed three times and mounted (SignalStain^®^ Mounting Medium, Cell Signaling Technology, #14177). The fluorescence images of P2RX4–SNAP-tag constructs and hCRC organoids were captured using a Leica TCS SP5 confocal laser scanning microscope (Leica Microsystems, Mannheim, Germany) with LAS AF (Leica Application Suite Advanced Fluorescence) software (version 1.8.1, build 1390). Imaging was performed using an HCX PL APO lambda blue oil-immersion objective (63.0×/NA 1.40). The confocal pinhole was set to 2 Airy units, and bidirectional scanning was used. An optical zoom factor of 4–6× was applied. Images were acquired at 12-bit depth.

### For the P2RX4–SNAP-tag constructs, *z*-stacked images were captured using a step size of 0.5 µm using the following settings: Hoechst signal was excited with a 405-nm diode laser (25% power) and detected between 415 and 472 nm with a photomultiplier tube (PMT) detector. The gain was set to 800 V, and the digital signal offset was −1. Wheat germ agglutinin (WGA), Alexa Fluor 488-conjugate (Thermo Fisher Scientific, #W11261), was excited with an argon laser (23%) and detected between 498 and 545 nm with a hybrid (HyD) detector. SNAP-Cell TMR-Star signal was excited with a 561-nm diode-pumped solid-state (DPSS) laser (10%) and detected between 565 and 780 nm with a hybrid (HyD) detector. For 2D image visualization, data were uploaded in ImageJ [v1.53e, National Institutes of Health (NIH), Bethesda, MD, USA] using the Bio-Formats Converter plugin. Representative *z*-stacks were visualized in grayscale or colored overlay. For figure visualization, brightness and contrast were adjusted uniformly for all images using ImageJ (NIH). In a 12-bit image (0–4095 grayscale range), the lower threshold was set to 100 and the upper threshold to 3,000. These adjustments were applied consistently across all images without altering the original pixel intensity values. Lateral projections were established using the orthogonal views function for stacks in ImageJ (NIH) from the full *z*-stacks.

### For 3D reconstruction and video rendering, the same raw image files were converted using the Imaris File Converter to the Imaris-supported format. The converted files were then uploaded into Imaris for further visualization and analysis.

*Tumor organoid culture and treatment*

Colon tumor organoids were cultured as previously described [32]. hCRC organoids were cultured in advanced Dulbecco’s modified Eagle’s medium/F12 containing RspoI, Noggin, B27, NAC, hEGF (PeproTech/Thermo Fisher Scientific, #AF-100-15), A83_01 (Tocris/Bio-Techne, Wiesbaden, Germany; #2939) and SB202190 (Sigma-Aldrich, Merck, Darmstadt, Germany; #S7067).

Human PDAC organoids were cultured in PancreaCult Organoid Media (Human) (StemCell Technologies, Vancouver, Canada; #100-0781) supplemented following the manufacturer’s protocol.

*Organoid reseeding capacity and organoid collection*

For analyzing the organoid reseeding capacity, hCRC and hPDAC organoids were seeded in equivalent numbers for each treatment condition. After 48 h, the organoids were treated as indicated in the figure legends for a further 48 h with 5-fluorouracil (20 µm for CRC lines and 10 µm for PDAC lines; Sigma-Aldrich, #F6627-5G), BAY-1797 (0.1 µm; Merck-Millipore, #SML2817-5M) or their combination and compared with DMSO (control; Sigma-Aldrich, #D8418-100ML). The reseeding capacity of treated organoids was compared with the reseeding capacity of DMSO-treated control organoids, which was set at 100%. Organoids were then collected, dissociated by pipetting them up and down 15 times using a 10-µl pipette tip placed on a 1,000-µl filter pipette tip, and then treated for approximately 20 min with Accutase (Sigma-Aldrich, #A6964). Single cell state was visually confirmed. Then cells were counted using Trypan Blue (Thermo Fisher Scientific, #15250061) and counted in technical duplicates (with two measurements per duplicate) using an automated cell counter (Corning, Wiesbaden, Germany; #6749). From each technical replicate, 40,000 viable single cells were plated three times in 50 µl Matrigel drops (Corning, #356231) to reduce the counting error associated with technical replication. hCRC organoids were cultured in advanced Dulbecco’s modified Eagle’s medium/F12 containing RspoI, Noggin, B27, NAC, hEGF (PeproTech/Thermo Fisher Scientific, #AF-100-15), A83_01 (Tocris/Bio-Techne, #2939) and SB202190 (Sigma-Aldrich, #S7067), and the media were changed once after 7 days. Sphere formation was analyzed after 14 days. Therefore, 4× images were taken three times in similar regions of each drop (per condition 6 drops comprising a total of 18 images).

Image quantification was performed using ImageJ software (NIH). A custom macro was established to standardize size cut-off and background subtraction and contrast enhancement using the following commands: macro "Reseeding capacity [Q]" {run("8-bit"); run("Subtract Background...", "rolling = 100 light"); run("Enhance Contrast...", "saturated = 0.35"); setAutoThreshold("Otsu no-reset"); //run("Threshold..."); setThreshold(178, 240, "raw"); run("Analyze Particles...", "size = 500-Infinity display include summarize add"). Organoid sphere size and number were extracted. The number of outgrowing spheres from 6 drops per condition were averaged and compared with the number in DMSO-treated control within each biological replicate. Each organoid line was analyzed in three independent experiments.

*Gene set enrichment analysis* (*GSEA*)

GSEA was performed using the fgsea R package (RRID:SCR_020938; version 1.18.0) as reported previously (<https://www.biorxiv.org/content/10.1101/060012v3>) [66]. Genes with base mean less than 10 were removed before they were ranked based on the log2 fold change in ascending order. The ranked gene list was compared against MSigDB (RRID:SCR_016863; version 7.4) using the ‘fgsea’ function with default settings. Normalized enrichment scores (NES) and adjusted *p* values were used for subsequent analyses.

The GSVA R package (RRID:SCR_021058; version 1.40.1) was used to determine gene set enrichment at the single sample level [62]. In brief, raw read counts were converted to log2 RPKM using the ‘rpkm’ function of the limma package (RRID:SCR_010943; version 3.48.3), and the ‘gsva’ function was used to calculate enrichment of the Hallmark signatures from MSigDB using default settings. Differentially expressed signatures were determined using the Bayesian method using the ‘Bayes’ function in the limma package. The top 20 signatures based on adjusted *p* value were used to extract sample- or context­specific signatures and were visualized as heat maps using the Complex­Heatmap R package (RRID:SCR_017270; version 2.8.0).

**Supplementary Figures**

**
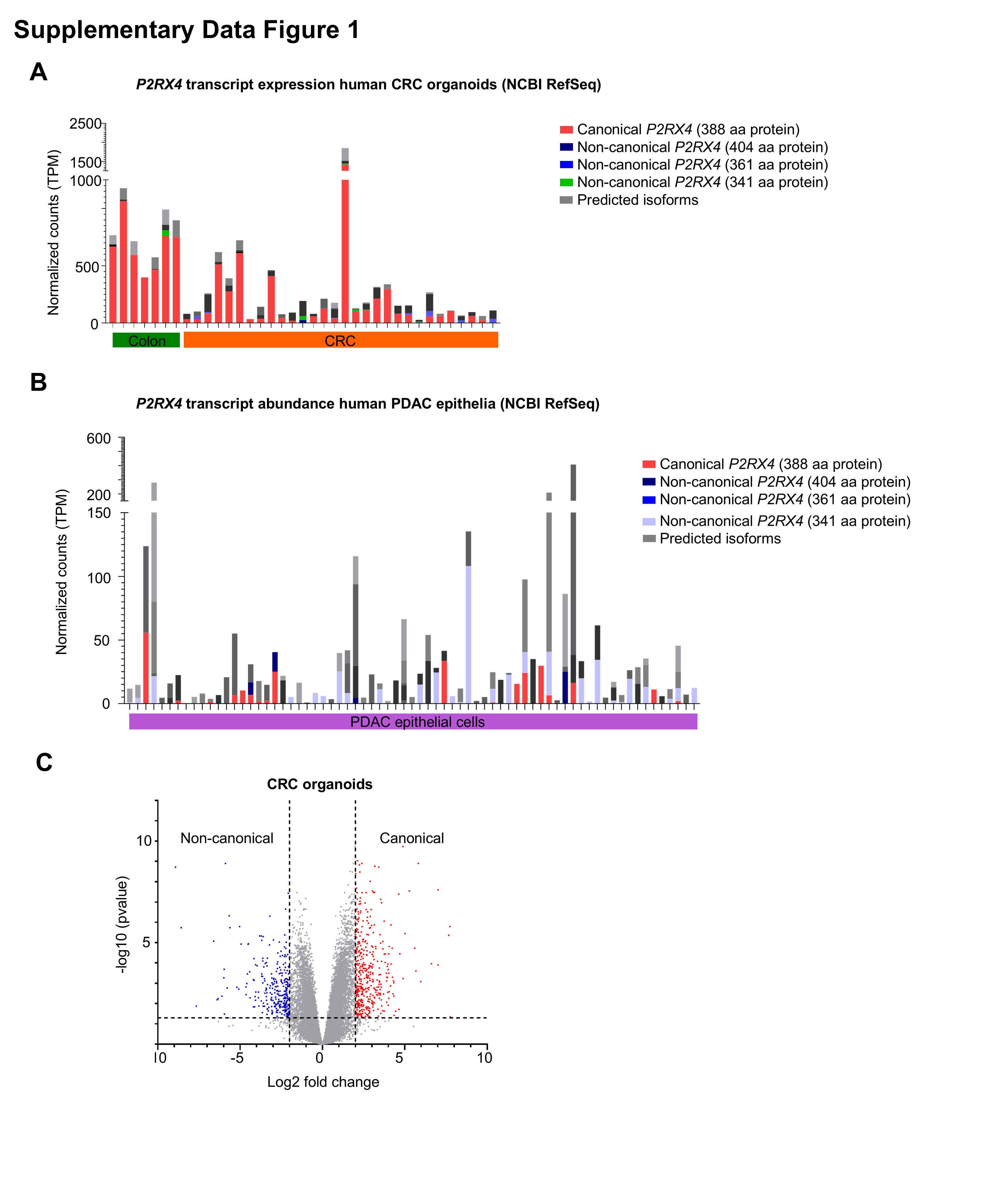
**

**Figure S1.** **Additional characterization of *P2RX4* transcript expression in human colorectal and pancreatic cancer.** (A) Bulk RNA sequencing*-*based *P2RX4* transcript expression (reference database: NCBI RefSeq annotation 110) in human colon and colorectal cancer organoids. Each bar represents one patient-derived (tumor) organoid. Grey shading indicates computationally predicted *P2RX4* transcripts. (B) Bulk RNA sequencing*-*based *P2RX4* transcript expression (reference database: NCBI RefSeq annotation 110) in data from [43], comprising tumor cells isolated by laser capture microdissection from fresh frozen primary human pancreatic adenocarcinoma. Each bar represents one patient sample. Grey shading indicates computationally predicted *P2RX4* transcripts. (C) Volcano plot of differentially expressed genes comparing patient-derived human colorectal cancer organoid lines expressing the canonical *P2RX4* transcript (*n* = 7) with the non-canonical *P2RX4*-expressing lines (*n* = 23). Genes were considered significantly regulated with an adjusted *p* value less than 0.05 and a log2 fold change greater than 2. A total of 432 upregulated (red) and 289 downregulated (blue) genes in the canonical group compared with the non-canonical group were identified.


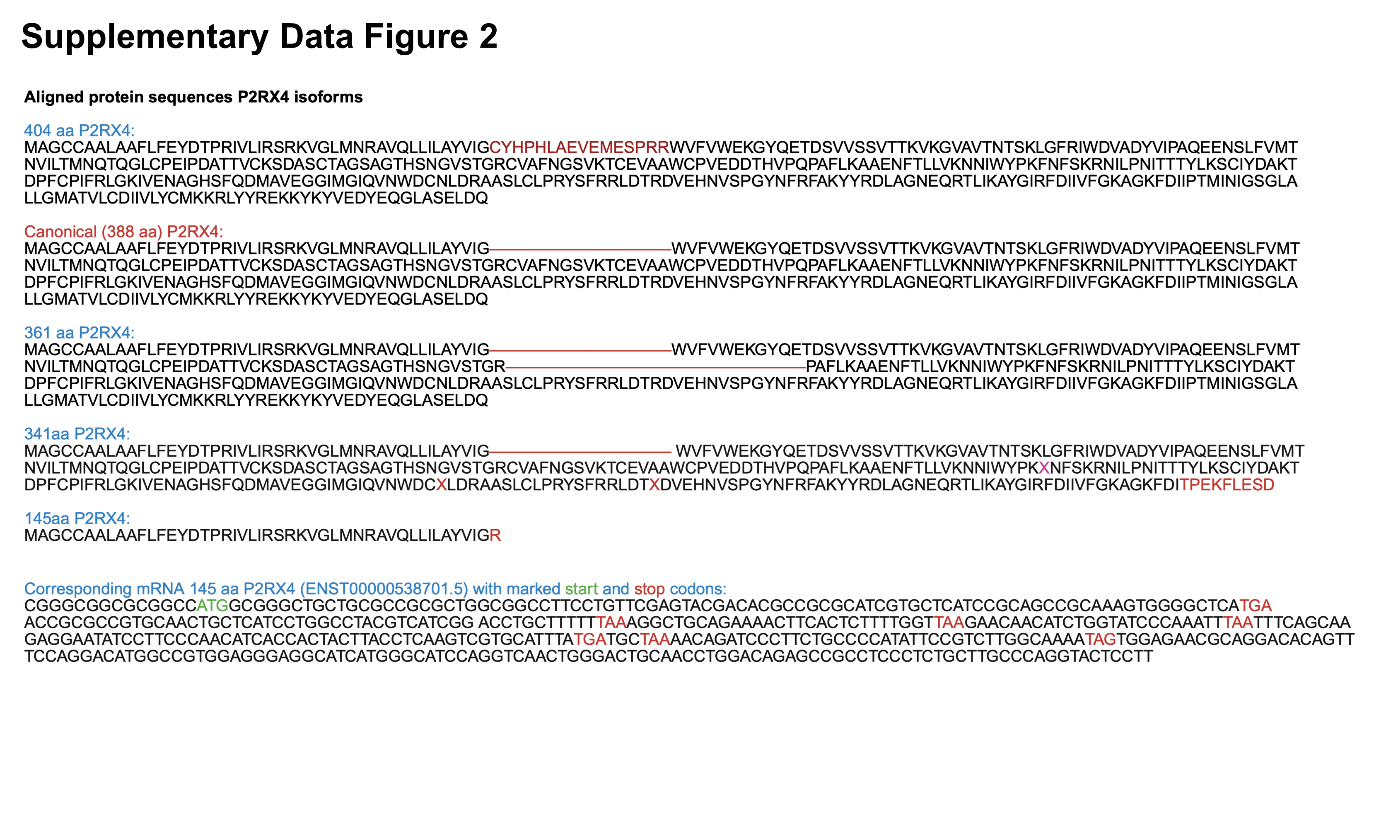


**Figure S2. Protein and mRNA sequences of human P2RX4 isoforms.** Protein sequences resulting from alternatively spliced *P2RX4* transcripts are shown. Variant regions relative to the longest sequence in the 404-aa acid isoform are highlighted in red. For the 145-aa isoform (ENST00000538701.5), the corresponding mRNA sequence is shown. The start codon is highlighted in green, and premature stop codons are highlighted in red.


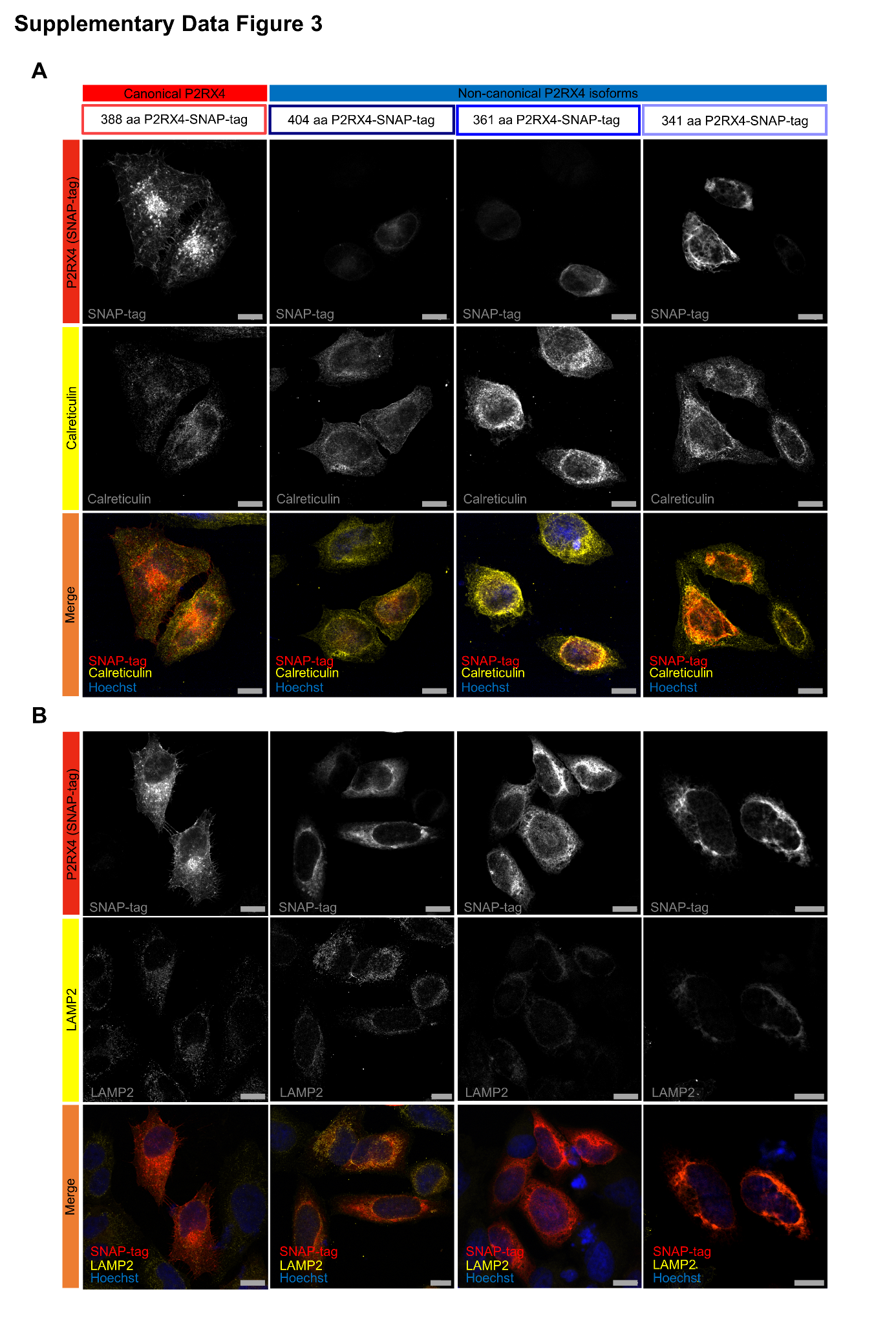


**Figure S3. P2RX4–SNAP-tag fusion constructs in the endoplasmic reticulum and lysosomal compartment.** Representative confocal microscopy images. HeLa cells were transfected with *P2RX4–*SNAP-tag constructs and labelled after 1 day with the TMR-Star reagent for SNAP-tag constructs. The cells were then fixed, permeabilized, and counterstained with (A) calreticulin (endoplasmic reticulum marker) and Hoechst (nuclear marker) or (B) LAMP2 (lysosomal marker) and Hoechst. Scale bars: 10 µm. The Hoechst channel is displayed only in the merged images.


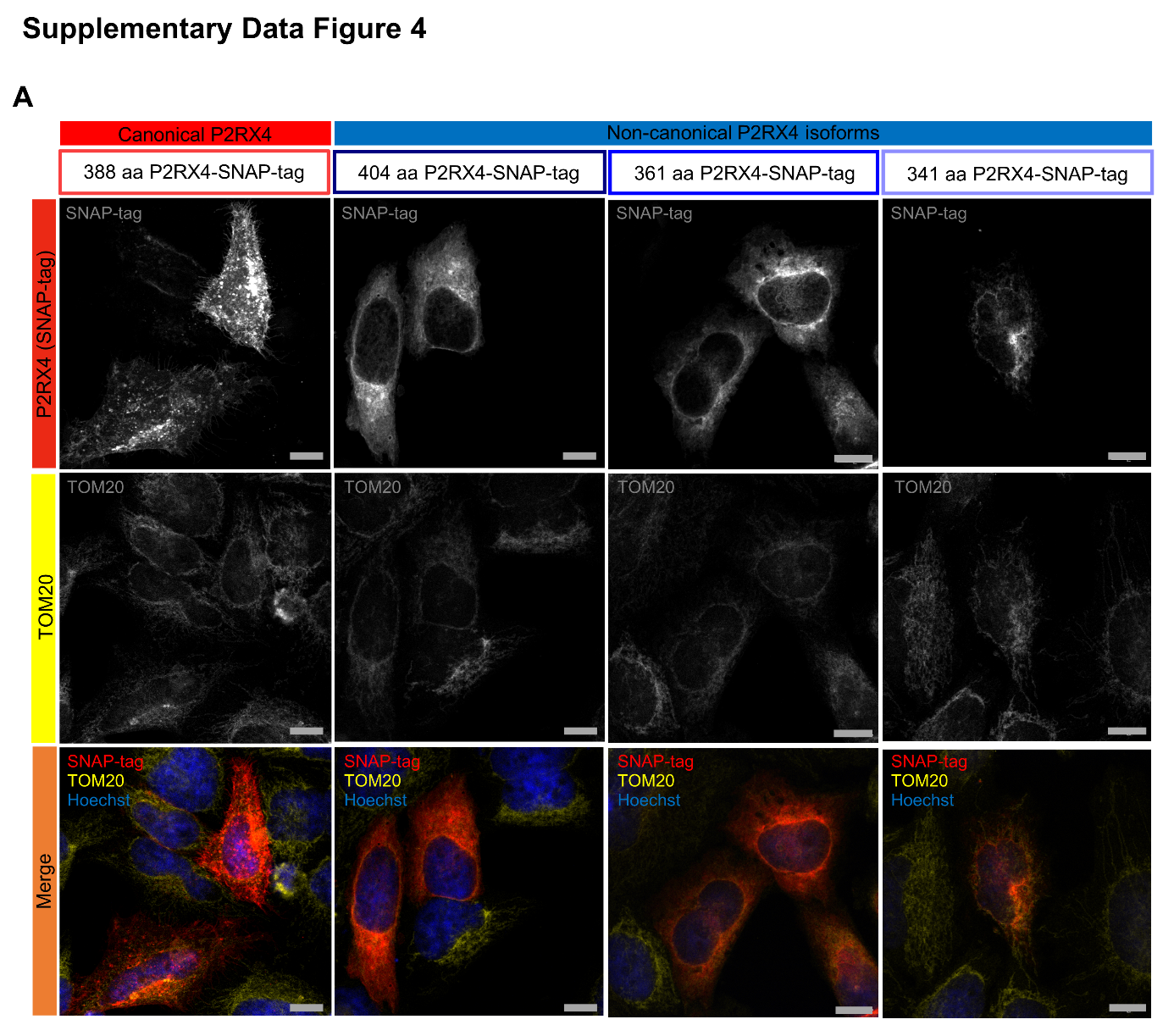


**Figure S4. P2RX4–SNAP-tag fusion constructs and mitochondria.** Representative confocal microscopy images. HeLa cells were transfected with *P2RX4*–SNAP-tag constructs and labelled after 1 day with the TMR-Star reagent for SNAP-tag constructs. The cells were then fixed, permeabilized, and counterstained with TOM20 (mitochondrial marker) and Hoechst (nuclear marker). Scale bars: 10 µm. The Hoechst channel is displayed only in the merged images.


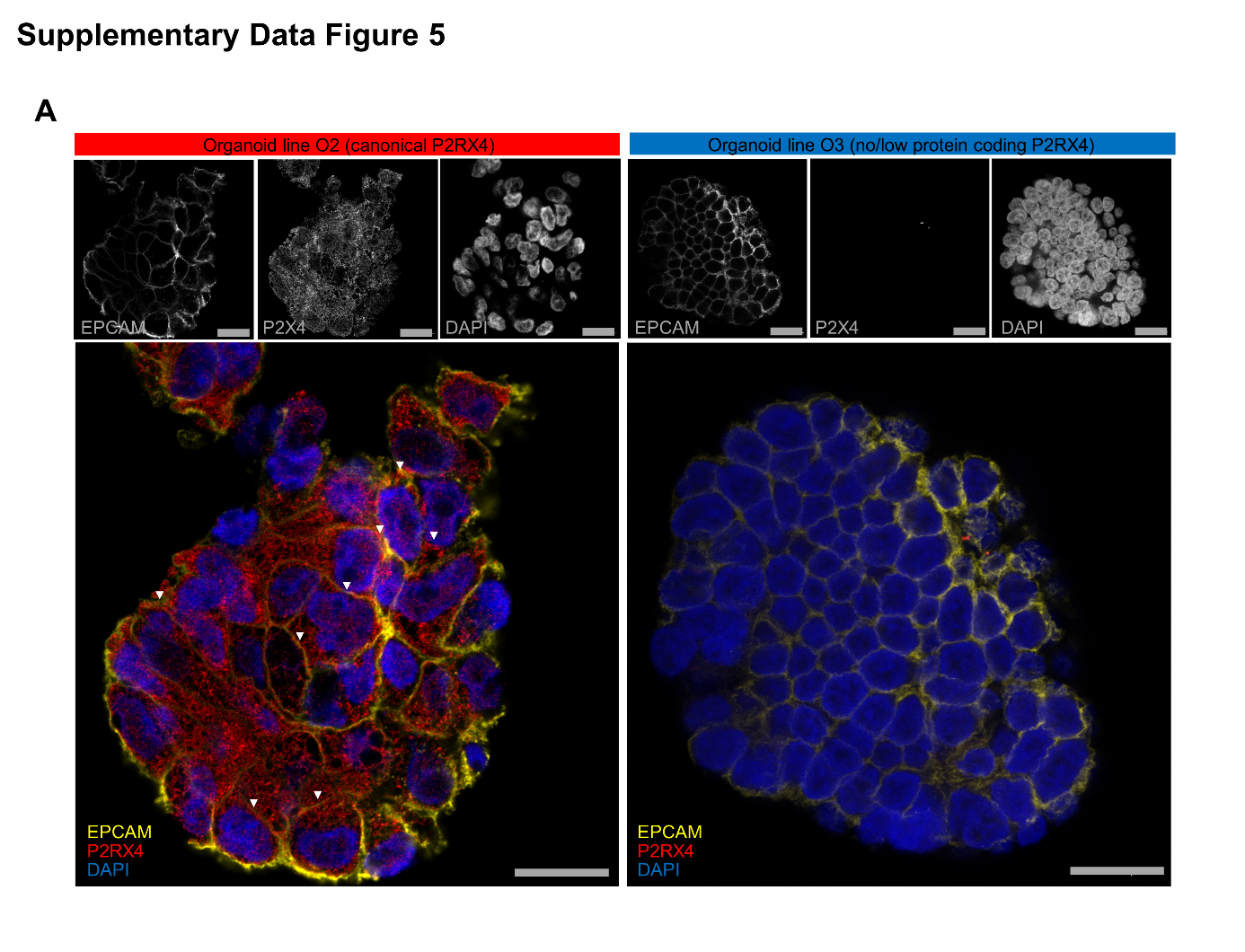


**Figure S5. P2RX4 expression in additional human colorectal cancer organoids.** Representative confocal microscopy images. Human colorectal cancer organoids expressing the canonical *P2RX4* transcript (O2, left) or low/no protein coding *P2RX4* transcripts (O3, right) were stained for P2RX4 and EPCAM and counterstained with DAPI. Scale bars: 10 µm. Arrowheads indicate areas of interest with P2RX4 and EPCAM overlap, indicating membrane expression.
